# Oncolytic adenovirus coexpressing interleukin-12 and decorin overcomes Treg-mediated immunosuppression inducing potent antitumor effects in a weakly immunogenic tumor model

**DOI:** 10.18632/oncotarget.13972

**Published:** 2016-12-10

**Authors:** Eonju Oh, Il-Kyu Choi, JinWoo Hong, Chae-Ok Yun

**Affiliations:** ^1^ Department of Bioengineering, College of Engineering, Hanyang University, Seongdong-gu, Seoul 133-791, Korea; ^2^ Department of Medical Oncology, Dana-Farber Cancer Institute, Harvard Medical School, Boston, MA 02215, USA

**Keywords:** oncolytic adenovirus, IL-12, decorin, TGF-β, Treg

## Abstract

Interleukin (IL)-12 is a potent antitumor cytokine. However, immunosuppressive tumor microenvironments containing transforming growth factor-β (TGF-β) attenuate cytokine-mediated antitumor immune responses. To enhance the efficacy of IL-12-mediated cancer immunotherapy, decorin (DCN) was explored as an adjuvant for overcoming TGF-β-mediated immunosuppression. We designed and generated a novel oncolytic adenovirus (Ad) coexpressing IL-12 and DCN (RdB/IL12/DCN). RdB/IL12/DCN-treated tumors showed significantly greater levels of interferon (IFN)-γ, tumor necrosis factor-α, monocyte chemoattractant protein-1, and IFN-γ-secreting immune cells than tumors treated with cognate control oncolytic Ad expressing a single therapeutic gene (RdB/DCN or RdB/IL12). Moreover, RdB/IL12/DCN attenuated intratumoral TGF-β expression, which positively correlated with reduction of Treg cells in draining lymph nodes and tumor tissues. Furthermore, tumor tissue treated with RdB/IL12/DCN showed increases infiltration of CD8^+^ T cells and proficient viral spreading within tumor tissues. These results demonstrated that an oncolytic Ad co-expressing IL-12 and DCN induces a potent antitumor immune response via restoration of antitumor immune function in a weakly immunogenic murine 4T1 orthotopic breast cancer model. These findings provide new insights into the therapeutic mechanisms of IL-12 plus DCN, making it a promising cancer immunotherapeutic agent for overcoming tumor-induced immunosuppression.

## INTRODUCTION

Cancer immunogene therapy is a promising alternative to conventional cancer therapy [1–3]. Cancer immunogene therapy focuses on efficient transfer of immune stimulatory genes such as chemokines, costimulators, and cytokines into tumor cells to induce antitumor immune responses [4–6]. A critical hurdle to successful cancer immunogene therapy is tumor-induced immunosuppression, which attenuates the potency of immunotherapeutics by anergy or tolerance.

Interleukin (IL)-12 is one of the most effective and promising cytokine for cancer immunotherapy. IL-12, which is secreted by activated macrophages and dendritic cells (DCs), facilitates T helper type 1 (Th1) differentiation and enhances the cytotoxicity of natural killer (NK) cells and cytotoxic T lymphocytes (CTLs) [7–9]. Preclinical evaluation of IL-12 shows induction of potent antitumor immunity in murine tumor models [10, 11]. However, clinical trials of recombinant IL-12 cytokine therapy do not yield satisfactory results due to tumor-induced immunosuppression and the transient nature of systemically administered IL-12. The tumors of patients in the clinical trials were composed of heterogeneous tumor cell populations that actively recruited or produced immunosuppressive factors, generating a highly immunosuppressive tumor microenvironment [9, 12–15]. Therefore, a novel strategy is required to improve the therapeutic efficacy of IL-12 in clinical environments. We previously demonstrated that cancer-specific expression and amplification of the IL-12 gene mediated by oncolytic adenovirus (Ad) maintains IL-12 expression at therapeutic doses for a prolonged period in murine melanoma models, resulting in potent antitumor efficacy [16–20].

Transforming growth factor-β (TGF-β) and regulatory T (Treg) cells are major contributors to the formation of immunosuppressive networks in tumor tissues that attenuate the potency of immunotherapeutics. TGF-β suppresses activation, maturation, and differentiation of immune cells such as CTLs, NK cells, and DCs [21]. TGF-β is integral to the maintenance and generation of immunosuppressive Treg cells that inhibit the antitumor immune functions of tumor-specific CD8*^+^* and CD4*^+^* T cells by cell-cell contact and production of immunosuppressive cytokines such as IL-10 or TGF-β [22, 23]. These immunosuppressive attributes are frequently observed in clinical tumors and inhibit the induction of effective antitumor immune responses [24, 25]. Weakly immunogenic tumor models have these immunosuppressive attributes of clinical tumors, making them useful for evaluating anticancer immunotherapeutics.

Decorin (DCN), a prototype member of a small leucine-rich proteoglycan family, is a ubiquitous component of the extracellular matrix (ECM) that regulates diverse functions through interaction with ECM components. DCN suppresses the biological activity of TGF-β by preventing TGF-β binding to its receptor [26–30]. DCN inhibits primary tumor growth and metastasis by decreasing TGF-β-induced immunosuppression [30]. Thus, inhibition of TGF-β expression in combination with immune stimulatory cytokines could induce antitumor immunity by overcoming tumor-mediated immunosuppression.

In this study, we generated an oncolytic Ad co-expressing IL-12 and DCN (RdB/IL12/DCN). The purpose was to suppress TGF-β-mediated immunosuppression in tumor microenvironments for enhanced induction of IL-12-mediated antitumor immune responses. In a weakly immunogenic murine breast cancer model, RdB/IL12/DCN elicited a potent antitumor effect by restoring antitumor immune function in a tumor milieu. Oncolytic Ad-mediated suppression of TGF-β expression in tumor tissues correlated with enhanced induction of antitumor immune responses as evidenced by enhanced infiltration of T cells into tumor tissue treated with RdB/IL12/DCN compared with a cognate control oncolytic Ad expressing a single therapeutic gene (RdB/DCN or RdB/IL12). In addition, we demonstrated that oncolytic Ad-mediated DCN expression enhanced the distribution of oncolytic Ad within tumor tissues, contributing to efficient transgene expression and the antitumor efficacy of RdB/IL12/DCN.

## RESULTS

### Expression of IL-12 and DCN by RdB/IL12/DCN

**Figure 1:**
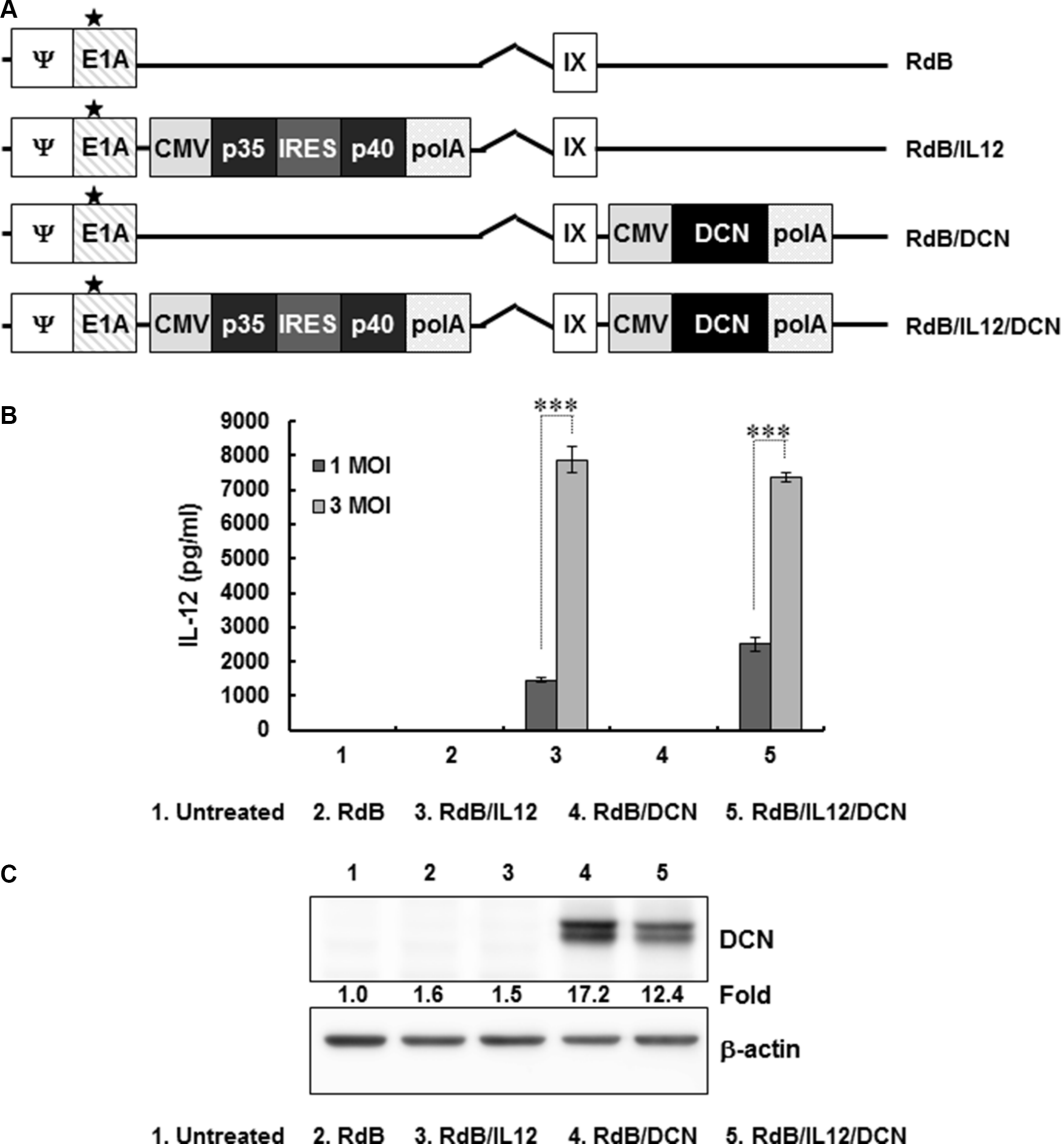
Characterization of oncolytic adenovirus (Ad) vectors expressing interleukin (IL)-12 and/or decorin (DCN) (**A**) Schematic representation of the genomic structures of oncolytic Ads. RdB has genes for mutated E1A and lacks E1B19 kD, E1B55 kD, and E3 genes. RdB/IL12 and RdB/DCN has genes for IL-12 in the E1 and DCN in the E3 region of RdB. RdB/IL12/DCN has genes for IL-12 in the E1 and DCN in the E3 region of RdB. Asterisk, mutation in the Rb binding site of E1A. (**B**, **C**) IL-12 and DCN expression in Ad-permissive U343 cells after infection with RdB, RdB/IL12, RdB/DCN, or RdB/IL12/DCN. Cell supernatants were harvested at 48 hr after infection and IL-12 expression was quantified by ELISA. Representative western blot of DCN using lysates harvested at 48 hr after infection. ELISA data are mean ± SD of triplicate experiments. ***P < 0.001.

To construct an oncolytic Ad co-expressing IL-12 and DCN, IL-12 and DCN genes were placed in the E1 and E3 region of oncolytic Ad, RdB, respectively (Figure 1A). To assess IL-12 expression mediated by the Ad, U343 cells were treated with RdB, RdB/IL12, RdB/DCN, or RdB/IL12/DCN at multiplicity of infection (MOI) 1 or 3. IL-12 secretion was dose-dependently elevated in cancer cells infected with either RdB/IL12 or RdB/IL12/DCN (Figure 1B; *P* < 0.001). DCN was analyzed by western blot. Cancer cells treated with RdB/DCN or RdB/IL12/DCN efficiently expressed DCN, whereas no expression was observed in the RdB-treated or RdB/IL12-treated groups (Figure 1C). These results demonstrated that IL-12 and DCN were efficiently expressed by RdB/IL12/DCN.

### Antitumor efficacy of RdB/IL12/DCN

Most types of cancer are weakly immunogenic due to antigen masking or overall immunosuppression [24, 25]. The therapeutic efficacy of IL-12-mediated cancer gene therapy is markedly attenuated in a weakly immunogenic 4T1 tumor model [31, 32]. TGF-β is the most potent immunosuppressive cytokine at inhibiting IL-12-induced antitumor immune responses by interfering with the IL-12 signaling pathway essential to interferon (IFN)-γ production. This effect results in the formation of an immunosuppressive network in tumors [33]. TGF-β inhibits the proliferation, differentiation, and function of macrophages, T cells, B cells, and NK cells [34–37]. Previously, we demonstrated that a DCN-expressing oncolytic Ad suppresses TGF-β expression in keloid fibroblasts [38]. We hypothesized that oncolytic Ad-mediated DCN expression would ameliorate TGF-β-induced immunosuppression in a tumor microenvironment, leading to enhanced induction of the antitumor immune response mediated by IL-12.

**Figure 2:**
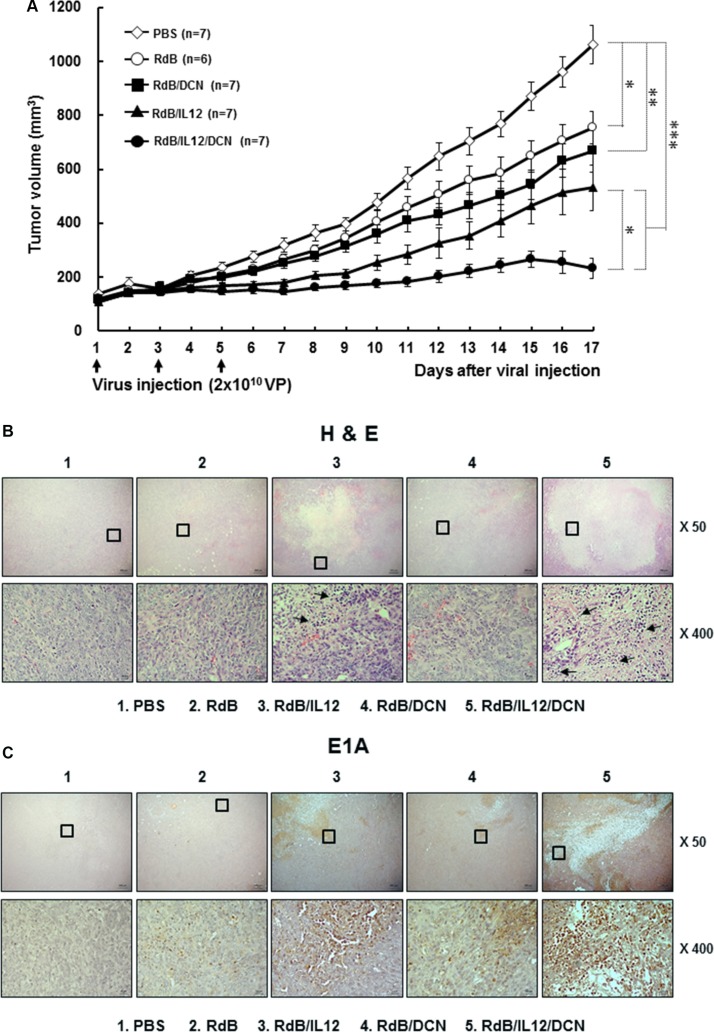
Antitumor effect of oncolytic Ads in an orthotopic breast cancer model (**A**) Established orthotopic 4T1 tumors were injected with RdB (open circles), RdB/DCN (filled squares), RdB/IL12 (filled triangles), or RdB/IL12/DCN (filled circles) at 2 × 10^10^ VP on days 1, 3, and 5, with PBS control (open diamonds). Tumor growth was monitored daily. Arrow, treatment administration. Data are mean ± SD of tumor volume by group (*n* = 6–7 mice). *P < 0.05, **P < 0.01, or ***P < 0.001. (**B** and **C**) Histology and immunohistochemistry of tumor tissues treated with PBS, RdB, RdB/IL12, RdB/DCN, or RdB/IL12/DCN. Tumor tissues were collected from mice at 6 days after final treatment. Paraffin sections were stained with hematoxylin and eosin (H&E) or anti-Ad E1A. Arrows, immune cell infiltration into tumor tissues. Images are representatives of results from three independent experiments. Original magnification: × 50 with × 400 magnification of the boxed area.

To assess the therapeutic potential of RdB/IL12/DCN in a weakly immunogenic 4T1 orthotopic breast cancer model, tumor-bearing mice were injected intratumorally with phosphate-buffered saline (PBS), RdB, RdB/IL12, RdB/DCN, or RdB/IL12/DCN. Tumors treated with PBS grew rapidly and average tumor volume reached 1065.6 ± 71.3 mm*^3^* by day 17 after initial treatment (Figure 2A). Inhibition of tumor growth was 29.3% for treatment with RdB (754.3 ± 59.7 mm*^3^*), 50.2% with RdB/IL12 (531.7 ± 85.0 mm*^3^*), 37.3% with RdB/DCN (668.8 ± 80.1 mm*^3^*), and 78.2% with RdB/IL12/DCN (233.1 ± 36.4 mm*^3^*) compared with PBS-treated control tumors (*P* < 0.05 for RdB, *P* < 0.01 for RdB/DCN, *P* < 0.001 for RdB/IL12 and RdB/IL12/DCN versus PBS). Both of the IL-12-expressing oncolytic Ads (RdB/IL12 and RdB/IL12/DCN) showed similar tumor growth inhibition up to day 9 after initial treatment. In RdB/IL12-treated tumors, significant tumor regrowth was observed, however, at later time periods. In contrast, RdB/IL12/DCN-treated tumors continued to show tumor growth inhibition during later time periods, suggesting that oncolytic Ad-mediated expression of DCN functioned as an adjuvant to IL-12 and enhanced the antitumor efficacy of oncolytic Ad (*P* < 0.05, RdB/IL12/DCN versus RdB/IL12 by 17 days).

To further assess antitumor efficacy mediated by RdB/IL12/DCN, tumor tissues were examined by histology and immunohistochemistry. A reduction was observed in viable tumor cells along with extensive necrotic regions in tumors treated with RdB/IL12 or RdB/IL12/DCN compared with tumors treated with PBS, RdB, or RdB/DCN (Figure 2B). RdB/IL12/DCN-treated tumors showed larger necrotic areas and more immune cell infiltration than RdB/IL12-treated tumors. This result implied that intratumoral expression of DCN facilitated induction of the cytokine-mediated antitumor immune response. In addition, higher expression of E1A, a viral replication marker, was observed in RdB/IL12/DCN-treated sections compared with RdB/IL12-treated sections (Figure 2C). This result suggested that intratumoral expression of DCN enhanced viral distribution and replication in tumor tissues. These results demonstrated that RdB/IL12/DCN induced potent and prolonged tumor growth inhibition by enhancing immune cell infiltration and viral dispersion within the tumor bed.

### Upregulation of IL-12, DCN, IFN-γ, TNF-α, and MCP-1 by RdB/IL12/DCN

**Figure 3:**
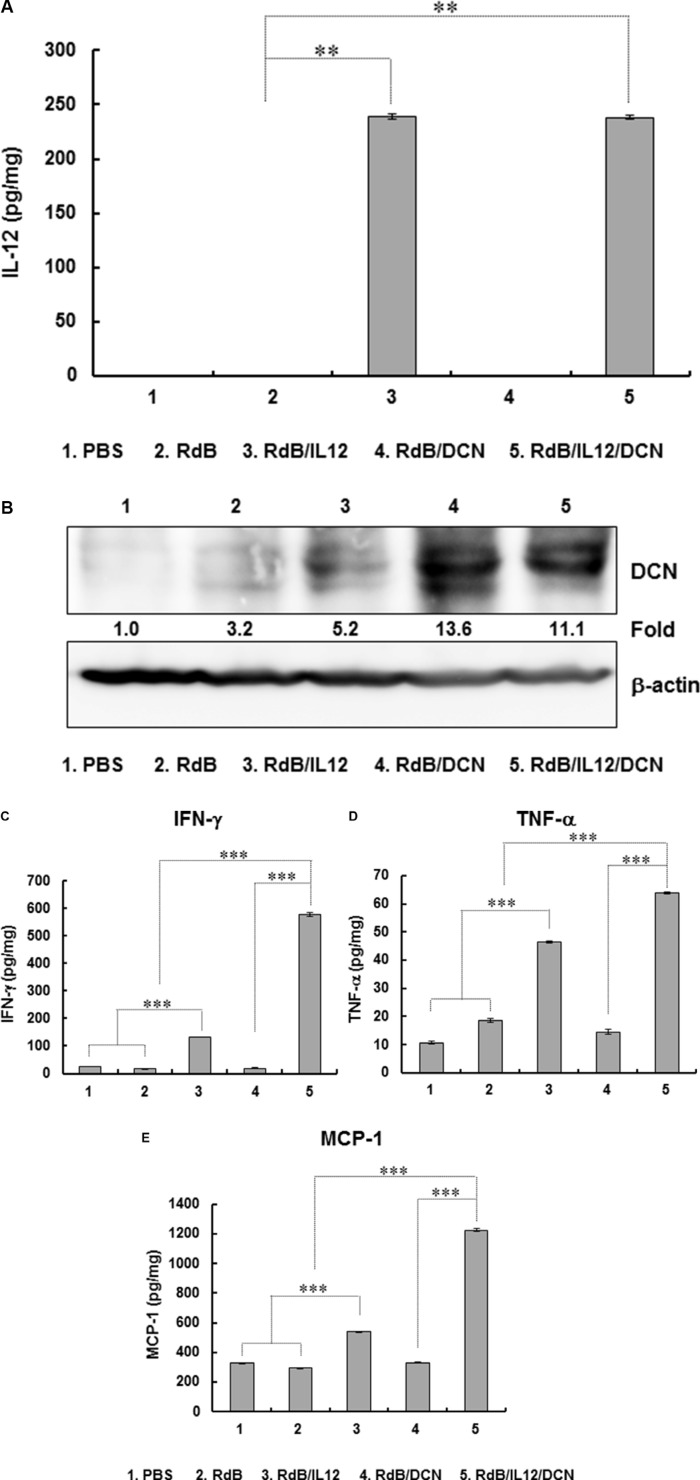
Expression of IL-12, DCN, IFN-γ, TNF-α, and MCP-1 in tumor tissues Tissues were obtained at 6 days after final viral treatment for quantitation of (**A**) IL-12, (**B**) DCN, (**C**) IFN-γ, (**D**) TNF-α, and (**E**) MCP-1. Experiments were in triplicate and repeated three times (A, C, D, E) DCN expression is representative of three independent experiments (B) Data points are mean expression ± SD for IL-12, IFN-γ, TNF-α, and MCP-1 for each tumor. **P < 0.01 or ***P < 0.001.

To evaluate the effect of oncolytic Ad-mediated immune-modulation of gene expression in tumor microenvironments, intratumoral cytokines were measured in tumor tissues. IL-12 levels were not detectable in tumors treated with PBS, RdB, or RdB/DCN (Figure 3A). However, tumors treated with RdB/IL12 or RdB/IL12/DCN showed high IL-12 levels (239.0 ± 3.0 pg/mg and 238.0 ± 2.0 pg/mg, respectively; *P* < 0.01). In addition, RdB/DCN or RdB/IL12/DCN-treated tumors exhibited significantly higher DCN expression than those treated with either RdB or RdB/IL12 (Figure 3B). These results implied that RdB/IL12/DCN expressed both therapeutic genes at high levels in tumor tissues.

IL-12 induces a Th1 immune response by promoting secretion of IFN-γ and tumor necrosis factor (TNF)-α from T and NK cells [59–61]. Therefore, we examined the expression of IFN-γ and TNF-α in tumor tissues treated with oncolytic Ads. RdB/IL12/DCN-inoculated tumors showed significantly higher levels of IFN-γ (579.1 ± 6.0 pg/mg) than tumors treated with PBS (23.9 ± 0.1 pg/mg), RdB (16.0 ± 0.2 pg/mg), RdB/DCN (19.4 ± 0.4 pg/mg), or RdB/IL12 (131.1 ± 0.7 pg/mg) (*P* < 0.001) (Figure 3C and 3D). Likewise, RdB/IL12/DCN-treated tumors exhibited significantly higher TNF-α expression (63.7 ± 0.3 pg/mg) than those treated with PBS (10.6 ± 0.4 pg/mg), RdB (18.6 ± 0.8 pg/mg), RdB/DCN (14.4 ± 0.8 pg/mg), or RdB/IL12 (46.4 ± 0.4 pg/mg) (*P* < 0.001). Of interest, the expression of both IFN-γ and TNF-α in tumor tissues treated with RdB/IL12/DCN was significantly greater than tumors treated with RdB/IL-12 (*P* < 0.001), although no significant difference was observed in IL-12 expression between tumors treated with the two oncolytic Ads (Figure 3A). These results suggested that IL-12-induced Th1 immune responses were enhanced by concurrent intratumoral expression of DCN.

Intratumoral expression of IFN-γ or TNF-α induces monocyte chemoattractant protein-1 (MCP-1) production, which enhances T cell infiltration into tumor tissues [39–41]. RdB/IL12/DCN-treated tumors exhibited significantly higher expression of MCP-1 (1224.4 ± 11.1 pg/mg) than tumors treated with PBS (328.0 ± 3.0 pg/mg), RdB (295.5 ± 3.4 pg/mg), RdB/DCN (329.6 ± 2.9 pg/mg), or RdB/IL12 (537.9 ± 3.9 pg/mg) (Figure 3E). A strong positive correlation was seen for expression of MCP-1, IFN-γ, and TNF-α (*P* < 0.001). These results suggested that intratumoral expression of Th1 cytokines (IFN-γ and TNF-α) and the T cell-recruiting chemokine MCP-1 were significantly enhanced by co-expression of IL-12 and DCN.

### Efficient induction of tumor-specific immunity by RdB/IL12/DCN

**Figure 4:**
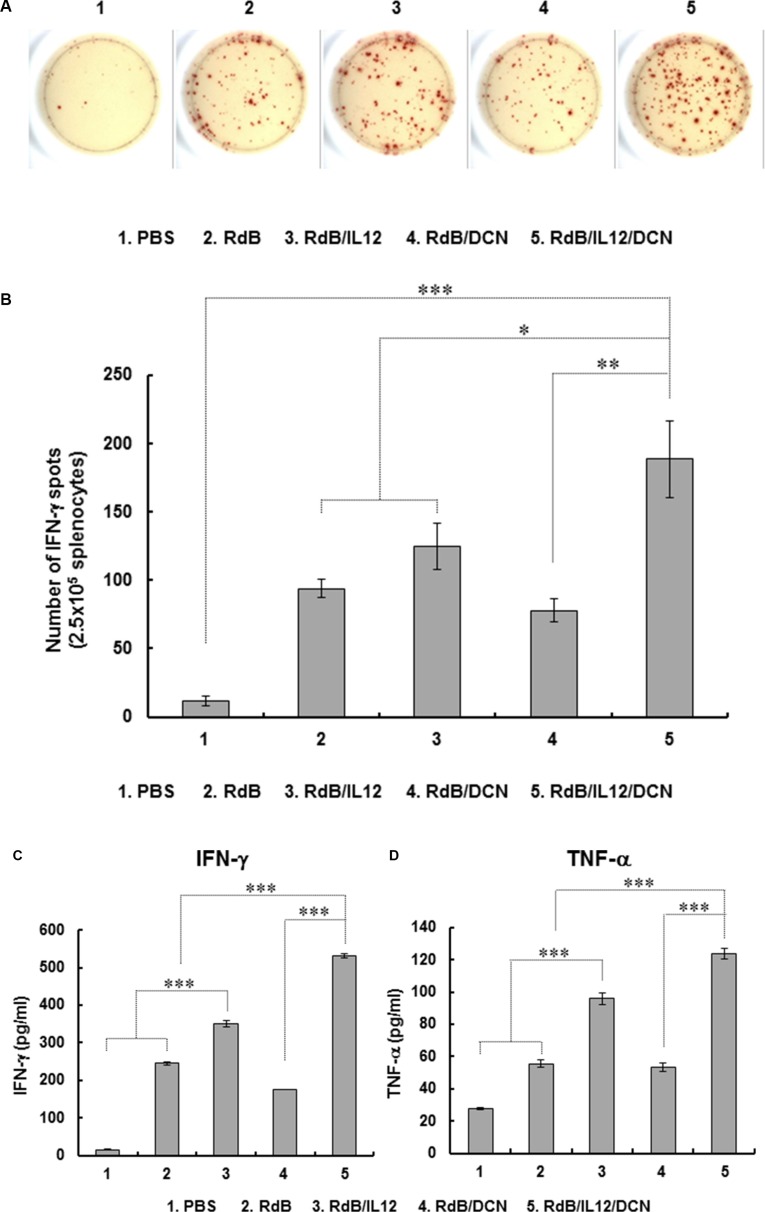
Assessment of tumor-specific immunity Splenocytes were collected from mice treated with PBS, RdB, RdB/IL12, RdB/DCN, or RdB/IL12/DCN at 6 days after final treatment and co-incubated with pre-irradiated 4T1 cells for 1 day. Assays were IFN-γ ELISPOT. (**A**) Spot-forming cell response. Images are representatives of results from three independent experiments. (**B**) Number of spots for 2.5 × 10^5^ splenocytes. Values are mean spot number ± SD for triplicates representative of three independent experiments. *P < 0.05, **P < 0.01, or ***P < 0.001. (**C**, **D**) Quantification of IFN-γ and TNF-α from tumor-specific immune cells. Splenocytes were co-cultured with pre-irradiated 4T1 cells for 2 days. IFN-γ and TNF-α were evaluated in co-cultured supernatants by cytometric bead array (CBA) mouse inflammation kits. Data points are mean ± SD of triplicates representative of three independent experiments. ***P < 0.001.

To assess the tumor-specific immune response mediated by oncolytic Ads, splenocytes were harvested from tumor-bearing mice at 6 days following last treatment and co-cultured with irradiated 4T1 cells. Splenocytes were evaluated for IFN-γ-secreting immune cells by IFN-γ ELISPOT. The number of IFN-γ-secreting immune cells was markedly elevated in RdB/IL12/DCN-treated mice compared with mice treated with PBS, RdB, RdB/IL12 or RdB/DCN (Figure 4A). Furthermore, IFN-γ-producing immune cells were recovered from RdB/IL12/DCN-treated mice significantly more frequently (188.7 ± 28.2) than from mice treated with PBS (11.7 ± 3.5), RdB (94.0 ± 2.6), RdB/IL12 (124.7 ± 17.2), or RdB/DCN (26.3 ± 8.9) (Figure 4B; *P* < 0.001 versus PBS, *P* < 0.05 versus RdB and RdB/IL12, *P* < 0.01 versus RdB/DCN). To further examine tumor-specific immunity driven by oncolytic Ads, IFN-γ and TNF-α were assessed in culture supernatants of splenocytes co-cultured with irradiated 4T1 cells. RdB/IL12/DCN-treated groups exhibited significantly higher IFN-γ and TNF-α than groups treated with PBS, RdB, RdB/IL12, or RdB/DCN (*P* < 0.001) (Figure 4C and 4D), suggesting that RdB/IL12/DCN induced a potent tumor-specific adaptive immune response.

### Downregulation of intratumoral TGF-β expression level by RdB/IL12/DCN

**Figure 5:**
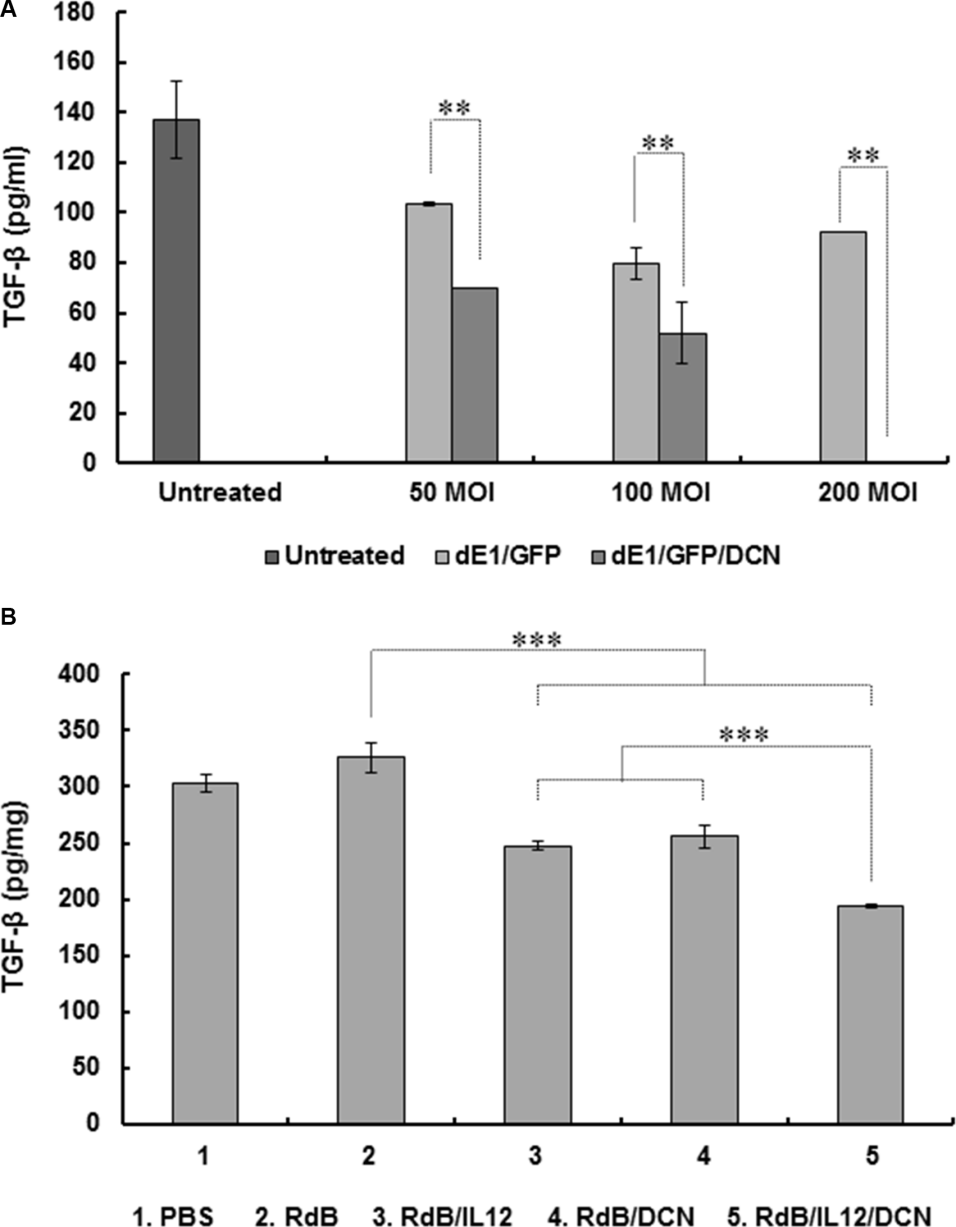
Inhibition of TGF-β expression by DCN-expressing Ad *in vitro* and *in vivo* (**A**) 4T1 cells were transduced with replication-incompetent dE1/GFP or dE1/GFP/DCN at indicated MOIs and TGF-β expression measured from culture supernatants at 48 hr after transduction using ELISA. Values are mean ± SD of triplicates representative of three independent experiments. **P < 0.01 compared with dE1/GFP. (**B**) Established 4T1 tumors were treated with PBS, RdB, RdB/IL12, RdB/DCN, or RdB/IL12/DCN, and harvested from mice at 5 days after final treatment. ELISA was performed to estimate TGF-β in tumor tissues. Data points are mean ± SD of triplicates representative of three independent experiments. ***P < 0.001 compared with RdB, RdB/IL12 or RdB/DCN.

To assess the effect of oncolytic Ads on expression of immunosuppressive TGF-β, we first evaluated the effect of green fluorescent protein (GFP) and DCN-expressing replication-incompetent Ad (dE1/GFP/DCN) on TGF-β expression in cancer cells. Treating 4T1 cells with dE1/GFP/DCN resulted in significant, dose-dependent inhibition of TGF-β expression compared to treatment with cognate control replication-incompetent Ad (dE1/GFP) (Figure 5A; *P* < 0.01). At 200 MOI, dE1/GFP/DCN completely suppressed TGF-β expression, implying that Ad-mediated DCN expression can efficiently inhibit TGF-β production in cancer cells.

To further assess the effect of oncolytic Ad-mediated DCN expression on suppression of TGF-β secretion in tumor microenvironments, expression of TGF-β in tumor tissues was assessed following administration of PBS control or oncolytic Ads. TGF-β expression was significantly attenuated in tumors treated with RdB/IL12, RdB/DCN, or RdB/IL12/DCN compared with tumors treated with RdB (Figure 5B), indicating that either transgene alone efficiently inhibited secretion of immunosuppressive TGF-β (*P* < 0.001). Importantly, RdB/IL12/DCN-treated tumors showed significantly attenuated TGF-β expression compared to tumors treated with RdB/IL12 or RdB/DCN (*P* < 0.001), suggesting that both IL-12 and DCN functioned as adjuvants for efficient suppression of TGF-β. These results suggested that co-expression of IL-12 and DCN efficiently downregulated immunosuppressive TGF-β expression in tumor tissue, leading to antitumor efficacy and tumor-specific immunity.

### Decreased Treg cells and enhanced accumulation of cytotoxic T cells in RdB/IL12/DCN-treated tumors

**Figure 6:**
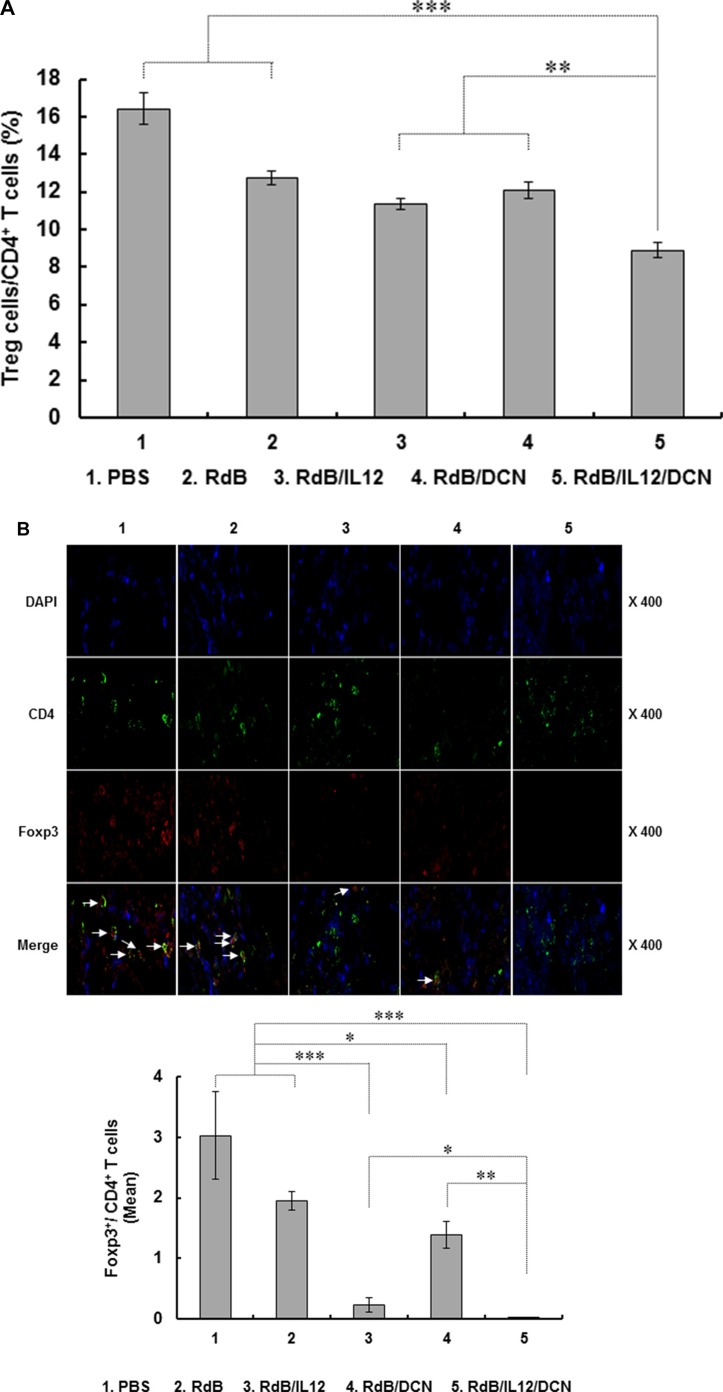

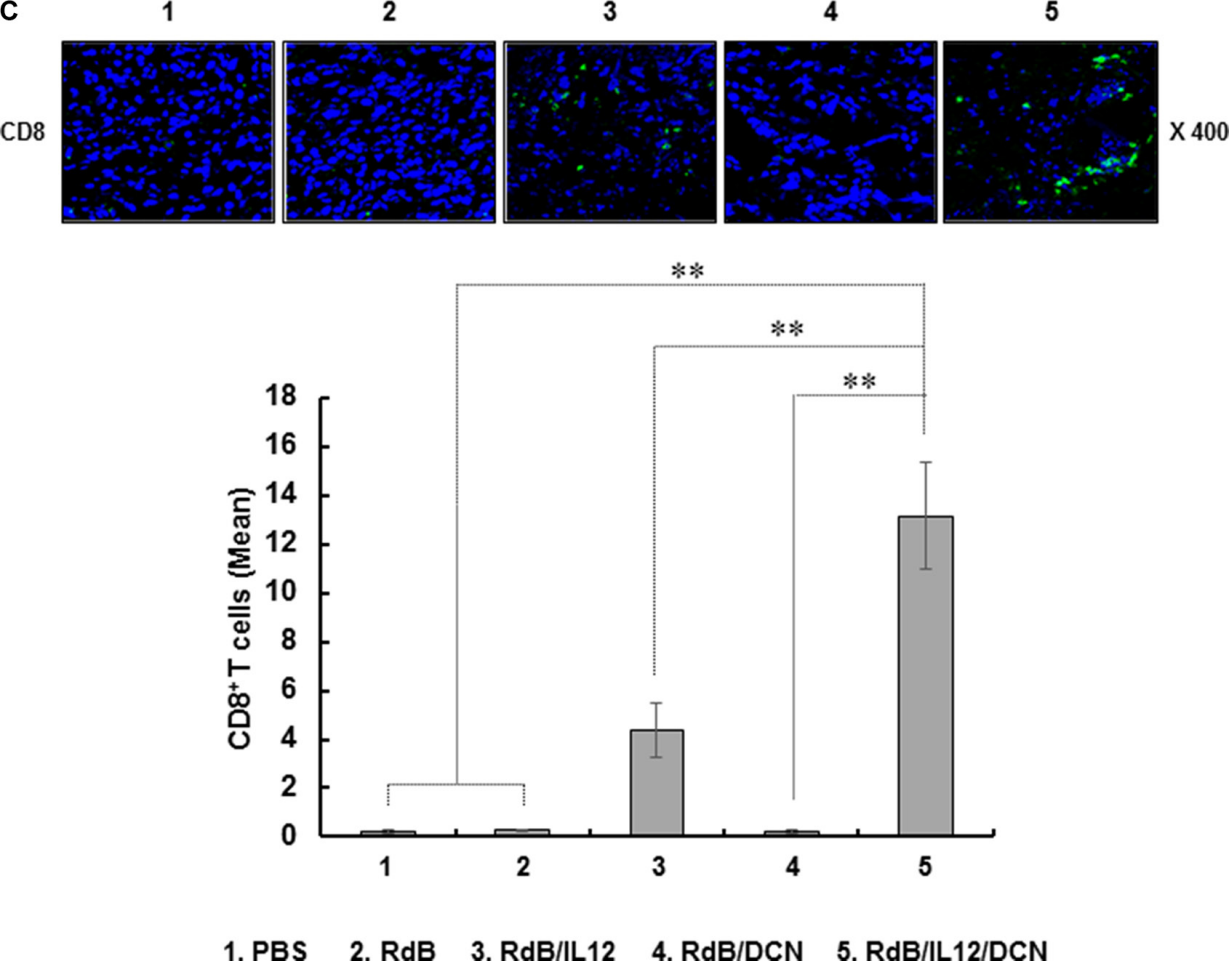
Proportion of Treg and quantification of CD8+ T cells DLNs and tumor tissues were collected from mice treated with PBS, RdB, RdB/IL12, RdB/DCN, or RdB/IL12/DCN at 6 days after final viral treatment. (**A**) Population of Treg cells in DLNs from mice were analyzed by flow cytometry. Gating was for CD4^+^ T cells and analysis for CD25^+^ and Foxp3^+^ cells. Data points are mean ± SD of triplicate experiments with at least three mice per group. Similar results were obtained from at least two separate experiments. **P < 0.01, ***P < 0.001. (**B**) Cryosections of tumor tissues stained with anti-CD4 (green) and anti-Foxp3 (red) monoclonal antibodies. Arrows, T cells co-expressing CD4 and Foxp3 in tumors. Ratio of Foxp3^+^/CD4^+^ T cells was assessed by ImageJ software. The relative mean intensity of CD4- or Foxp3-positive cells were quantified from three independent fields within microscope images for each experimental group. Data are representative of three independent experiments. *P < 0.05, **P < 0.01, or ***P < 0.001. (**C**) Cryosections of tumor tissues were incubated with antibody against CD8 (green). Mean ± SD of CD8^+^ T cells was quantified by ImageJ software. The mean intensity of three different images was quantified for each sample (CD8^+^ T cells/field). Images are representative of three independent experiments. Original magnification: × 400. **P < 0.01.

CD4*^+^*CD25*^+^*Foxp3*^+^* Treg cells are key contributors to TGF-β-mediated immunosuppression in tumor microenvironments [42, 43]. To assess the effects of oncolytic Ads on the CD4*^+^*CD25*^+^*Foxp3*^+^* Treg cell population, we examined draining lymph nodes (DLNs) from tumor-bearing mice by fluorescence-activated cell sorting (FACS). By gating for CD4*^+^* cells and analyzing expression of CD25 and Foxp3, we found that the Treg population was significantly reduced in DLNs from mice treated with RdB/IL12/DCN compared to mice treated with PBS, RdB, RdB/IL12, or RdB/DCN (Figure 6A; *P* < 0.001 versus PBS and RdB, *P* < 0.01 versus RdB/IL12 and RdB/DCN).

To assess intratumoral infiltration of Treg cells, tumor tissues were examined by immunofluorescence double staining with anti-CD4 and anti-Foxp3 antibodies (Abs). Foxp3*^+^* Treg cells were attenuated following treatment with RdB/IL12 (*P* < 0.001), RdB/DCN (*P* < 0.05), or RdB/IL12/DCN (*P* < 0.001) compared to treatment with PBS or RdB (Figure 6B). More importantly, the ratio of Treg cells in the CD4*^+^* T cell population was lower in tumor tissues treated with RdB/IL12/DCN than in tissues treated with RdB/IL12 (*P* < 0.05) or RdB/DCN (*P* < 0.01). This result suggested that accumulation of immunosuppressive Treg cells in tumors was attenuated more by co-expression of IL-12 and DCN than by expressing either gene alone.

Accumulation of Treg cells correlates with reduced infiltration of CD8*^+^* T cells in tumors and poor prognosis for cancer patients [44–46]. To evaluate the density of tumor-infiltrating CD8*^+^* T cells following oncolytic Ad treatments, tumor tissues were assessed by immunohistochemistry using CD8-specific Abs. Markedly higher quantities of CD8*^+^* T cells were found in RdB/IL12/DCN-treated tumors compared with tumors treated with PBS, RdB, RdB/IL12 or RdB/DCN (*P* < 0.01) (Figure 6C), suggesting that the decreased population of Treg cells correlated with enhanced infiltration of CD8*^+^* T cells into tumor tissues. Collectively, these results demonstrated that an oncolytic Ad co-expressing IL-12 and DCN alleviated TGF-β-mediated immunosuppression and reduced infiltration of immunosuppressive Treg cells in tumor microenvironments, facilitating IL-12-mediated T cell antitumor immunity.

## DISCUSSION

Immunogene therapy focuses on regulating the antitumor immune response using immune stimulatory factors such as cytokines and costimulatory factors to activate and recruit immune cells to tumor tissues [47, 48]. In clinical tumor microenvironments, interaction between cancer, immune, and stromal cells contributes to the formation and maintenance of an immunosuppressive network that promotes tumor growth and attenuates the efficacy of immunogene therapy [3, 49, 50]. Therefore, combining immune modulators with factors capable of amending the immunosuppressive tumor microenvironment is critical for inducing an optimal antitumor immune response.

IL-12 induces a potent antitumor immune response by promoting Th1 differentiation of CD4*^+^* T cells and activating CTL- and NK cell-mediated cytotoxicity [51]. However, systemic administration of IL-12 induces severe side effects in both preclinical animal models and clinical trials [52–55]. To attenuate IL-12-mediated systemic toxicities, the IL-12 gene has been incorporated into viral [56–58] and nonviral [8, 59, 60] vectors [59, 61–63], resulting in improved safety and therapeutic efficacy. In particular, cytokine-expressing oncolytic Ads demonstrate potent therapeutic outcomes as viral replication amplifies expression of cytokines, tumor cell lysis, and release of tumor-associated antigens *in situ* [16–20].

Tumor-induced immunosuppression is another critical hurdle of IL-12-mediated cancer immunotherapy in clinical trials. Clinical tumors are often weakly immunogenic or nonimmunogenic due to minimal expression of tumor antigens, downregulated expression of MHC class I molecules and antigen presentation capability, T cell anergy, and immunosuppression; this leads to low immunotherapy efficacy [24, 25]. In tumor-induced immunosuppression, TGF-β overexpression and accumulation of Treg cells in tumor tissues are integral components of tumor immune evasion mechanisms [21, 42, 64]. To overcome immunosuppression in tumor microenvironments mediated by Treg cells and TGF-β, we generated an oncolytic Ad co-expressing IL-12 and DCN. Our aim was to enhance IL-12-mediated induction of the antitumor immune response through DCN-mediated downregulation of TGF-β activity in a weakly immunogenic murine 4T1 orthotopic breast cancer model that mimics clinical tumors.

Earlier reports demonstrated that the core structure of DCN is composed of 10 central leucine-rich repeats that suppress TGF-β activity by preventing receptor binding. DCN also negatively regulates the stability of critical downstream effectors in the insulin-like growth factor receptor I signaling pathway, indirectly inhibiting TGF-β receptor signaling [30, 65–67]. Consistent with these reports, DCN-expressing Ads attenuated TGF-β expression *in vitro* and *in vivo*, suggesting that Ad-mediated DCN expression can functionally block TGF-β expression (Figure 5A and 5B). Further, in weakly immunogenic tumors, treatment with oncolytic Ad coexpressing IL-12 and DCN elicited more antitumor effects than treatment with an oncolytic Ad expressing IL-12 or DCN alone (Figure 2A). Of note, RdB/IL12/DCN-treated mice had larger necrotic regions and viral dispersion in tumors than mice treated with RdB/DCN or RdB/IL12 (Figure 2B and 2C). These results are in good agreement with our previous report that DCN-expressing oncolytic Ad induces apoptosis and ECM degradation, leading to enhanced viral dispersion in tumor tissues [26, 68]. RdB/IL12/DCN-treated tumor tissues showed more infiltration of immune cells than tissues treated with RdB/IL12. This result suggested that DCN-mediated degradation of ECM and downregulation of TGF-β activity facilitated infiltration of immune cells into tumors for enhanced induction of an antitumor immune response. Together, these results demonstrated that co-expression of IL-12 and DCN by a single oncolytic Ad vector induced a potent antitumor effects by inducing extensive necrosis, efficient infiltration of immune cells, and proficient viral distribution in tumor tissues.

RdB/IL12/DCN-treated tumors exhibited similar expression of IL-12 as tumors treated with RdB/IL12 (Figure 3A). However, both IFN-γ and TNF-α expression was significantly higher in RdB/IL12/DCN-treated tumor tissues (Figure 3C and 3D). Of note, the expression of TGF-β in RdB/IL12/DCN-treated tumor tissues was significantly lower than in tissues treated with RdB, RdB/IL12 or RdB/DCN, indicating that IL-12 and DCN may function as adjuvant to restore the immune stimulatory function of IL-12 and attenuate immunosuppressive TGF-β expression (Figure 5B). These findings are in good agreement with a previous report demonstrating that IL-12-activated human T cells are inhibited by TGF-β, which interferes with an IL-12-mediated signaling pathway [69].

Increased expression of Th1 cytokines (IFN-γ and TNF-α) and attenuation of TGF-β expression in tumor microenvironments positively correlated with enhanced tumor-specific adaptive immune responses. The evidence was the significantly elevated secretion of IFN-γ or TNF-α by tumor-specific immune cells of RdB/IL12/DCN-treated mice compared with RdB/IL-12-treated mice (Figure 4C and 4D). These observations were supported by the presence of markedly higher IFN-γ-secreting cells in the spleen of mice treated with RdB/IL12/DCN compared with that of RdB/IL12 (Figure 4A and 4B). These findings suggested that RdB/IL12/DCN may create a tumor microenvironment more favorable for activation and generation oftumor-specific immune cells through DCN-mediated amelioration of a immunosuppressive tumor network and IL-12-mediated upregulation of Th1 cytokines.

High expression of TGF-β in a tumor microenvironment suppresses DC differentiation and function, leading to generation of immature myeloid DCs that promote Treg cell proliferation [22, 70]. In addition, TGF-β promotes conversion of CD4*^+^*CD25*^−^* T cells into Treg cells [71–73]. Therefore, inhibition of TGF-β expression could restore antitumor immune function in a tumor milieu by reduction of the Treg cell population. Consistent with these reports, RdB/IL12/DCN-treated mice exhibited significantly fewer Treg cells in both DLN and tumor tissues compared with mice treated with RdB, RdB/IL12 or RdB/DCN. This finding positively correlated with TGF-β expression (Figure 5B), implying that oncolytic Ad-mediated suppression of TGF-β inhibited the accumulation of immunosuppressive Treg cells in DLN and tumor tissues (Figure 6A and 6B). These results suggested that oncolytic Ad-mediated expression of DCN ameliorated the immunosuppressive tumor microenvironment via downregulation of TGF-β expression and the Treg cell population, leading to potent induction of an antitumor immune response.

High MCP-1 levels, which can be caused by elevated Th1 cytokine expression, promote recruitment and activation of T cells, resulting in tumor regression [40, 41, 74, 75]. In good agreement with these reports, RdB/IL12/DCN-treated tumors, which exhibited the highest MCP-1 levels, showed the highest infiltration of CD8*^+^* T cells (Figures 3E and 6C). An increase in intratumoral MCP-1, IFN-γ, and TNF-α expression was well correlated with more infiltration of CD8*^+^* T cells into tumor tissues treated with oncolytic Ads. These results demonstrated that oncolytic Ad-mediated co-expression of IL-12 and DCN induced an antitumor immune response in a weakly immunogenic tumor by downregulation of immunosuppression, active expression of cytokines, and enhanced influx of immune effector T cells. This effect resulted in an effective IL-12-mediated Th1 antitumor immune response.

Collectively, these results demonstrated that combining IL-12 and DCN is a promising candidate strategy to induce a potent antitumor immune response for treating highly immunosuppressive clinical tumors by overcoming Treg-mediated immunosuppression.

## MATERIALS AND METHODS

### Cell lines and cultures

U343 (human glioma cell line) and 4T1 (murine breast carcinoma cell line) were from the American Type Culture Collection (ATCC, Manassas, VA, USA). U343 cells were cultured in high-glucose Dulbecco's Modified Eagle's Medium (DMEM, Gibco BRL, Grand Island, NY, USA) and 4T1 cells in RPMI-1640 (Gibco BRL) containing 10% fetal bovine serum (FBS, Gibco BRL). All cell lines were cultured at 37°C in a humidified atmosphere 5% CO_*2*_ and 95% air.

### Mice

Female BALB/c mice, 6- to 8-week-old, were from Charles River Japan, Inc. (Yokohama, Japan) and maintained in a laminar air-flow cabinet with specific pathogen-free conditions. All facilities were approved by the Association for Assessment and Accreditation of Laboratory Animal Care. All animal studies were performed according to the institutionally approved protocols of Hanyang University.

### Construction and generation of Ad

Replication-incompetent Ad expressing GFP) and/or DCN (dE1/GFP and dE1/GFP/DCN) was generated as described previously [26] (Supplementary Figure S1). To construct an oncolytic Ad co-expressing IL-12 at the E1 region and DCN at the E3 region, respectively, the pSP72-E3/DCN Ad E3 shuttle vector [26] was linearized and co-transformed with *Spe*I-digested RdB/IL12 Ad total vector [76] into *Escherichia coli* BJ5183, generating an RdB/IL12/DCN Ad total plasmid. Ads used in this study were propagated in 293 and purified by CsCl gradient centrifugation as described previously [77]. Ads were stored at −80°C until use. Numbers of viral particles (VPs) were calculated from optical density measurements at 260 nm (OD_*260*_), where absorbance 1 (OD_*260*_ = 1) was equivalent to 1.1 × 10*^12^* VP/ml.

### Quantification of IL-12 and DCN expression

U343 cells were plated onto 6-well plates at 1 × 10*^5^* cells/well, and infected with RdB, RdB/IL12, RdB/DCN, or RdB/IL12/DCN at MOI 1 or 3. At 48 hr after infection, supernatants were obtained. IL-12 was determined by ELISA according to manufacturer's instructions (IL-12 ELISA kit: Endogen, Woburn, MA, USA). DCN was determined by western blot analysis as previously described [76]. In brief, at 48 hr after infection of U343 cells with RdB, RdB/IL12, RdB/DCN, or RdB/IL12/DCN at MOI 3, proteins from cell extracts were separated by 10% sodium dodecyl sulfate-polyacrylamide gel electrophoresis and transferred to PVDF membranes (RPN 303F, Amersham, Arlington Heights, IL). Membranes were incubated with primary anti-DCN Ab (R&D Systems, Minneapolis, MN) or anti-β-actin Ab (Cell Signaling Technology, Beverly, MA), then horseradish peroxidase-conjugated secondary Ab (Cell Signaling Technology).

For evaluating IL-12 and DCN expression in tumor tissues, tissues were collected from mice treated with Ad at 6 days after final viral treatment. Tumor tissues were homogenized (ART-MICCRA D-8; ART modern Labortechnik, Munchen, Germany) in ice-cold RIPA buffer (Elipis Biotech, Taejeon, South Korea) with a proteinase inhibitor cocktail (Sigma-Aldrich). Homogenates were centrifuged in a high-speed microcentrifuge for 10 min and total protein was determined using BCA protein assay reagent kits (Pierce, Rockford, IL). Levels of IL-12 in the tumor tissue extract and serum were measured by ELISA (Supplementary Figure S3). DCN expression in the tumor tissue extract was assessed by western blot analysis. DCN expression was semiquantitatively analyzed using ImageJ software (version 1.50b; U.S. National Institutes of Health, Bethesda, MD).

### Quantification of TGF-β expression

For transduction, 4T1 cells were plated onto 6-well plates at 1 × 10*^5^* cells, then transduced with dE1/GFP or dE1/GFP/DCN at MOI 50, 100, or 200. At 48 hr after transduction, supernatants were collected. To remove endogenously produced TGF-β, medium was replaced with serum-free RPMI 1640 media 24 hr before harvest time points. TGF-β expression was determined by ELISA according to the manufacturer's protocol for TGF-β ELISA kits (R&D Systems). For assessing TGF-β expression in a tumor milieu, tumor tissues were collected from mice treated with PBS, RdB, RdB/IL12, RdB/DCN, or RdB/IL12/DCN at 5 days after final treatment. Total protein was prepared as described above and TGF-β was measured by ELISA. ELISA results were normalized to total protein concentration in each group and calculated as picograms per milligrams of total.

### Antitumor effects of oncolytic Ad co-expressing IL-12 and DCN

To compare antitumor effects of RdB, RdB/IL12, RdB/DCN, or RdB/IL12/DCN, an orthotopic breast cancer model was established by subcutaneously injecting 4T1 cells (1 × 10*^6^*) into fat pads of 6- to 7-week-old female BABL/c mice. When average tumor volume reached 110–120 mm*^3^*, mice were randomly sorted into groups (PBS, RdB, RdB/IL12, RdB/DCN, and RdB/IL12/DCN) with similar mean tumor volumes and treatment was initiated. The first treatment day was designated as day 1. Ad or PBS was administrated intratumorally (2 × 10*^10^* VP per tumor in 20 μL PBS with 1% DMSO) on days 1, 3, and 5. Tumor growth was monitored daily by electronic caliper measure as volume = 0.523 LW*^2^* (L = length of tumor, W = width).

### Quantification of MCP-1, IFN-γ, and TNF-α expression

Tumor tissues were harvested from mice treated with oncolytic Ad at 6 days after last viral treatment. MCP-1, IFN-γ, and TNF-α in tumor tissue extracts or serum (Supplementary Figure S3) were measured by cytometric bead array (CBA) mouse inflammation kits (BD Biosciences). Results were normalized to total protein concentration per tumor and calculated as picograms per milligram of total protein.

To measure IFN-γ and TNF-α released by tumor-specific immune cells, spleens were obtained aseptically from tumor-bearing mice at 6 days following last viral treatment. Unicellular splenocytes were prepared as previously described [19]. Prepared splenocytes (1.5 × 10*^6^*) were co-cultured with irradiated 4T1 (1.5 × 10*^5^* cells; 5 Gy) for 2 days in the presence of recombinant mouse IL-2 (100 units/mL, R&D Systems). Culture supernatants were harvested and analyzed using CBA mouse inflammation kits (BD Biosciences).

### IFN-γ ELISPOT assay in splenocytes

To assess tumor-specific IFN-γ-producing immune cell populations, IFN-γ ELISPOT assays were performed. Six days following last Ad injection, spleens were harvested aseptically from mice, and unicellular splenocytes prepared as previously described [19]. Prepared spleen cells (2.5 × 10*^5^*) were co-cultured with pre-irradiated 4T1 (5.0 × 10*^3^* cells; 5 Gy) for 24 hr in the presence of recombinant mouse IL-2 (100 units/mL). IFN-γ ELISPOT assays were performed according to the manufacturer's specifications for IFN-γ ELISPOT kits (BD Biosciences). Colored spots were quantitatively analyzed by a computer-based immunospot system (AID Elispot Reader System, Version 3.4, Autoimmun Diagnostika GmbH, Strassberg, Germany).

### Fluorescence-activated cell sorting analysis

For the assessment Treg cell populations by FACS, DLNs were harvested at 6 days following final viral treatment of 4T1 tumor-bearing mice. Cells were pretreated with saturating anti-CD16/CD32 Ab (Biolegend, San Diego, CA) in staining buffer (2% FBS, 0.02% sodium azide in PBS) to block cellular Fc receptors. Cells were stained extracellularly with peridinin chlorophyll protein-CY5.5-conjugated anti-CD4 Ab (BD Biosciences) and phycoerythrin-conjugated anti-CD25 Ab (eBioscience, San Diego, CA). Cells were then permeabilized with Foxp3 fixation/permeabilization buffer (eBioscience) according to the supplier's protocol and stained with allophycocyanin-conjugated anti-Foxp3 Ab (eBioscience). Samples were analyzed using a BD Biosciences BD FACScanto II flow cytometry analyzer and FACSDiva software (BD Biosciences).

### Histological and immunohistochemical analysis

Tumor tissues were harvested from mice at 6 days after final treatment and fixed in 10% formalin, processed for paraffin embedding, and cut into 5-mm sections. Sections were stained with hematoxylin and eosin (H&E) and examined by light microscopy. Tumor sections were also immunostained with rabbit anti-Ad E1A polyclonal Ab (Santa Cruz Biotechnology) to assess viral replication and spreading. Slides were counterstained with Meyer's hematoxylin. To detect lymphocytes, tumor tissues were dehydrated with 30% sucrose at 4°C, frozen in OCT compound (Sakura Finetec, Torrance, CA), and cut into 10-mm sections. Cryosections were incubated with rat anti-mouse CD4 monoclonal Ab (BD Biosciences), rat anti-mouse CD8 monoclonal Ab, or rabbit anti-Foxp3 monoclonal Ab (Abcam, Cambridge, UK). After incubating with primary Ab at 4°C overnight, sections were incubated with Alexa Fluor 488-conjugated anti-rat IgG (Invitrogen, Carlsbad, CA, USA) or Alexa Fluor 568-conjugated anti-rabbit IgG as secondary Abs for 1 hr. For counterstaining, samples were incubated with 4,6-diamidino-2-phenylindole (DAPI, Sigma-Aldrich) and semiquantitatively analyzed by ImageJ software.

### Statistical analysis

Data were expressed as mean ± standard deviation (SD). Statistical significance was determined by two-tailed Student *t*-test (SPSS 13.0 software; SPSS, Chicago, IL). *P < 0.05, **P < 0.01 and ***P < 0.001 were considered statistically significant.

## SUPPLEMENTARY MATERIALS FIGURES AND TABLES


