# Supplementary material for: Oncolytic adenovirus coexpressing interleukin-12 and decorin overcomes Treg-mediated immunosuppression inducing potent antitumor effects in a weakly immunogenic tumor model
Source: Oncotarget. 2016 Dec 10;8(3):4730–46. doi: 10.18632/oncotarget.13972 (PMC5354867; doi:10.18632/oncotarget.13972)
Supplement: Supplementary file 1 [file oncotarget-08-4730-s001.pdf]

Oncolytic adenovirus coexpressing interleukin-12 and decorin overcomes Treg-mediated immunosuppression inducing potent antitumor effects in a weakly immunogenic tumor model

Supplementary Materials

MATERIALS AND METHODS

Viral production

4T1 cells were seeded in a 12-well plate at 5×10^4 cells/well, and infected with RdB at MOI 0.5. At 4, 48, and 72 hr post-infection, both supernatant and cells were collected. The copy number of Ad genomes was measured by real-time quantitative PCR (TaqMan PCR detection; Applied Biosystems, Foster City, CA) as previously described [78] (Supplementary Figure S2).

Expression levels of CAR in 4T1 cells

The expression levels of coxsackievirus and adenovirus receptor (CAR) on the surface of 4T1 cells were determined by flow cytometry. Cells were harvested by trypsin-EDTA treatment and washed with PBS (pH 7.4) containing 1% FBS. Cells were then incubated with fluorescein isothiocyanate-conjugated anti-CAR Ab (Santa Cruz Biotechnology, Santa Cruz, CA, USA) at 4°C for 1 hr. Cells were washed two times with PBS containing 1% FBS. Flow cytometry was performed using the FACSCalibur analyzer (BD Biosciences, San Jose, CA) with Cell Quest software (BD Biosciences) (Supplementary Table S1).

Assessment of *in vivo* toxicity

To measure *in vivo* toxicity of each oncolytic Ads, mice were administrated intratumorally with 2×10^{10} VP of RdB, RdB/IL12, RdB/DCN, or RdB/IL12/DCN along with PBS as a control. Serum levels of aspartate aminotransferase (AST) and alanine transaminase (ALT) were then measured at 6 days post injection (Supplementary Table S2).

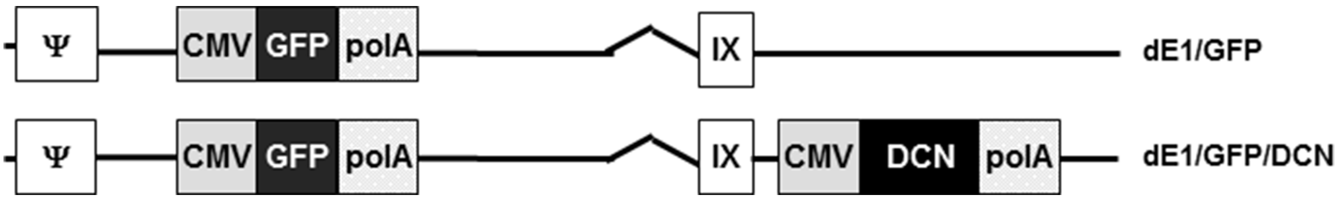

Supplementary Figure S1: Schematic representation of genomic structures of replication-incompetent DCN-expressing Ad. dE1/GFP, GFP gene in E1 region of replication-incompetent Ad (dE1). dE1/GFP/DCN, GFP and DCN genes into the E1 and E3 regions, respectively, of dE1.

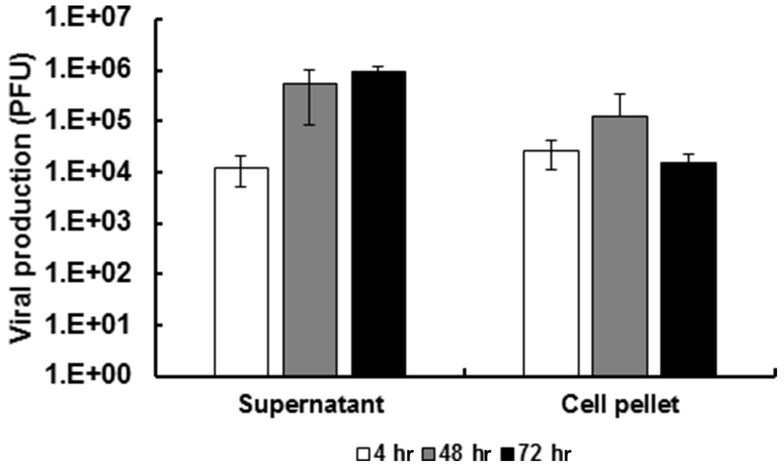

Supplementary Figure S2: Viral replication of Ad. 4T1 cells were infected with RdB at an MOI of 0.5. Cells and supernatant were sampled at 4, 48, and 72 hr post-infection and viral genomic copies were measured by real-time quantitative PCR. These experiments were conducted at least three times, and data shown are representative of these experiments. Data presented as mean ± SD.

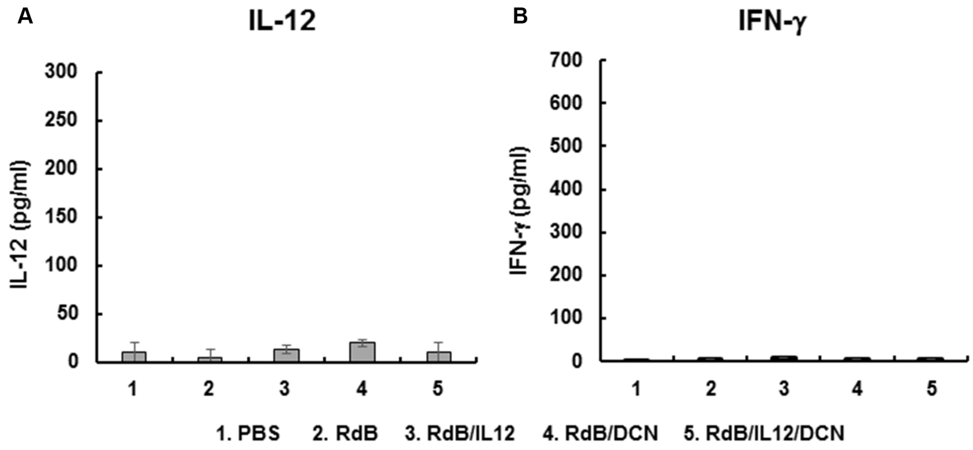

Supplementary Figure S3: Level of IL-12 and IFN-γ in serum. Serum was obtained at 6 days after final viral treatment for quantitation of (**A**) IL-12 and (**B**) IFN-γ. Each data point indicates mean ± SD of triplicates experiments.

Supplementary Table S1: Determination of CAR levels by FACS

Cell line	FACS (%)
4T1	0.17 ± 0.005

4T1 cells were incubated with fluorescein isothiocyanate-conjugated anti-CAR Ab. Data presented as the percentage of cells gated positive and determined in triplicate experiments

Supplementary Table S2: ALT and AST in serum

Group	AST (SGOT) (U/L)	ALT (SGPT) (U/L)
PBS	125.7 ± 35.8	27.7 ± 10.1
RdB	116.3 ± 34.6	21.7 ± 3.1
RdB/IL12	114.7 ± 25.7	28.3 ± 5.1
RdB/DCN	133.3 ± 36.7	19.3 ± 3.2
RdB/IL12/DCN	128.3 ± 70.2	27.7 ± 9.0

Serum was obtained at 6 days after final viral treatment for measurement of ALT and AST. Each data point indicates mean ± SD of triplicates experiments
